# Expression Profile of Multidrug Resistance Efflux Pumps During Intracellular Life of Adherent-Invasive *Escherichia coli* Strain LF82

**DOI:** 10.3389/fmicb.2020.01935

**Published:** 2020-08-17

**Authors:** Giulia Fanelli, Martina Pasqua, Bianca Colonna, Gianni Prosseda, Milena Grossi

**Affiliations:** Istituto Pasteur Italia, Dipartimento di Biologia e Biotecnologie “Charles Darwin”, Sapienza-Università di Roma, Rome, Italy

**Keywords:** efflux pumps, adherent invasive *Escherichia coli*, *Escherichia coli* pathogens, bacteria-host interactions, bacterial transmembrane complexes

## Abstract

Efflux pumps (EPs) are present in all living cells and represent a large and important group of transmembrane proteins involved in transport processes. In bacteria, multidrug resistance efflux pumps (MDR EPs) confer resistance to antibiotics at different levels and are deeply implicated in the fast and dramatic emergence of antibiotic resistance. Recently, several reports have outlined the great versatility of MDR EPs in exporting a large variety of compounds other than antibiotics, thus promoting bacterial adaptation to a wide range of habitats. In several bacterial pathogens, MDR EPs contribute to increase the virulence potential and are directly involved in the crosstalk with host cells. In this work, we have investigated the possible role of MDR EPs in the infectious process of the adherent-invasive *Escherichia coli* (AIEC), a group of pathogenic *E. coli* that colonize the ileal mucosa of Crohn disease (CD) patients causing a strong intestinal inflammation. The results we have obtained indicate that, with the exception of *mdtM*, all MDR-EPs encoding genes present in *E.coli* K12 are conserved in the AIEC prototype strain LF82. The analysis of MDR EP expression during LF82 infection of macrophages and epithelial cells reveals that their transcription is highly modulated during the bacterial intracellular life. Notably, some EP genes are regulated in a cell-type specific manner, strongly suggesting that their function is required for LF82 successful infection. AIEC are able to adhere to and invade intestinal epithelial cells and, importantly, to survive and multiply within macrophages. Thus, we further investigated the role of EPs specifically induced by macrophage environment. We present evidence indicating that deletion of *mdtEF* genes, encoding an MDR EP belonging to the resistance nodulation division (RND) family, significantly impairs survival of LF82 in macrophages and that the wild type phenotype can be restored by trans-complementation with functional MdtEF pump. Altogether, our results indicate a strong involvement of MDR EPs in host pathogen interaction also in AIEC and highlight the contribution of MdtEF to the fitness of LF82 in the macrophage environment.

## Introduction

Efflux pumps (EPs) are membrane protein complexes found in all living organisms. In many bacteria, including pathogens, EPs mediate the efflux of one or more antibiotics, thus strongly contributing to the development of multidrug resistance (MDR; Li et al., 2015; Du et al., 2018). EPs are usually present in the inner membrane as single-component transporters. In Gram-negative bacteria, they can form a tripartite structure spanning both membranes and consisting of an inner membrane protein, a periplasmatic adaptor protein and an outer membrane protein (Hinchliffe et al., 2013). On the basis of sequence similarity, transport function and energy source bacterial MDR EPs have been grouped into six families: ATP binding cassette (ABC), resistance nodulation division (RND), major facilitator superfamily (MFS), multidrug and toxic compound extrusion (MATE), small multidrug resistance (SMR), and proteobacterial antimicrobial compound efflux (PACE; Du et al., 2018). Recently, an additional family has been identified: the p-Aminobenzoyl-glutamate transporter (AbgT) family (Delmar and Yu, 2016). EPs use the proton motive force of the inner membrane as energy source. ABC EPs are an exception as they rely on ATP hydrolysis (Li et al., 2015; Du et al., 2018).

Multidrug resistance efflux pumps (MDR EPs) have been thoroughly studied because of their clinical relevance in bacterial infections (Li et al., 2015; Du et al., 2018). In recent years, it has become clear that the importance of MDR EPs goes beyond the efflux of antibiotics (Piddock, 2006; Alvarez-Ortega et al., 2013; Alcalde-Rico et al., 2016; Pasqua et al., 2019b) and involves several cellular functions. In particular, due to their ability to extrude a variety of different compounds, MDR EPs play a relevant role in the interactions of bacteria with plant and animal cells, in the maintenance of cellular homeostasis, in the detoxification of metabolic intermediates, and in cell-to-cell communication. Moreover, MDR EPs actively contribute to virulence of bacterial pathogens in plant and animals, including humans (Piddock, 2006; Alcalde-Rico et al., 2016). In opportunistic pathogens associated with cystic fibrosis, e.g., *Pseudomonas aeruginosa*, *Acinetobacter baumannii*, and *Stenotrophomonas maltophilia*, MDR EPs contribute to the export of quorum sensing molecules and favor the formation of biofilms (Alav et al., 2018; Pasqua et al., 2019b).

In some enteropathogens, MDR EPs, besides contributing to resistance to bile salts (Alvarez-Ortega et al., 2013; Leuzzi et al., 2015; Urdaneta and Casadesús, 2018), play a relevant role in the intracellular life of the bacterium. For example, in *Salmonella* Typhimurium MDR EPs are required for an efficient invasion and survival within macrophages and intestinal cells (Buckley et al., 2006; Bogomolnaya et al., 2013) while in *Listeria monocytogenes* they favor bacterial intracellular spread and tissue invasion through their capability to activate IFN-β production in infected mouse macrophages (Crimmins et al., 2008). Furthermore, it has been shown that, in *Staphylococcus aureus*, EPs contribute to the invasion of human epithelia and facilitate the persistence within staphylococcal-induced abscesses (Truong-Bolduc et al., 2015) and that, in *Mycobacterium tubercolosis*, an increased expression of MDR EP genes promotes bacterial replication in macrophages and survival in mouse models (Bianco et al., 2011).

In a recent study (Pasqua et al., 2019a), we were able to demonstrate that the MDR EmrKY EP significantly contributes to the survival of *Shigella flexneri* in macrophages. *S. flexneri* belongs to the *Escherichia coli* species and its invasive process is characterized by the capability to invade macrophages, where it multiplies and induces cell death (Schroeder and Hilbi, 2008; Pasqua et al., 2017). The bacteria released from dying macrophages invade neighboring enterocytes, where they rapidly lyse the vacuole and actively replicate. Invasion of macrophages and epithelial cells is a pathogenicity step also found in another group of enteropathogenic *E. coli*, the adherent and invasive *E. coli* (AIEC; Darfeuille-Michaud, 2002; Croxen et al., 2013). AIEC represent a pathotype associated with Crohn disease (CD), an inflammatory syndrome affecting the intestinal tract (Darfeuille-Michaud, 2002; Palmela et al., 2018; Shaler et al., 2019). AIEC strains do not express virulence factors typically found in other pathogenic *E. coli* and cluster within the *E. coli* B2 phylogenetic group whose members are mostly extraintestinal *E. coli* (Miquel et al., 2010). Despite the capability to invade the same host cells, AIEC and *Shigella* exhibit different intracellular survival strategies. AIEC replicate extensively in large vacuoles within macrophages without inducing host cell death (Glasser et al., 2001) and stimulate the production of large amount of TNF-α, leading to chronic inflammation. Moreover, in AIEC strains, the invasion of human intestinal epithelia occurs *via* a macropinocytosis-like process and involves the interaction of bacterial Type 1 pili with the CEACAM6 glycoprotein receptor, which is abnormally expressed in CD patients (Barnich et al., 2007). As opposed to *Shigella*, in epithelial cells, AIEC strains do not lyse the vacuole soon after invasion and replicate within late endosomes to escape cell autophagy (Lapaquette et al., 2010).

The different behavior of AIEC and *Shigella* within host cells has prompted us to investigate the expression of the MDR EPs of clinical strain LF82, considered as an AIEC prototype (Boudeau et al., 1999), during the invasion of epithelial cells and macrophages. The results we have obtained indicate that within these environments bacteria display a strong induction of several EPs, some of which are host-cell specific. As the extensive replication of LF82 in macrophage phagolysosomes is a critical step for the intracellular survival of the pathogen (Glasser et al., 2001; Demarre et al., 2019), we have focused on EPs highly expressed only within macrophages and have found that MdtEF, a MDR EP belonging to the RND family, significantly contributes to bacterial fitness in this environment.

## Materials and Methods

### Bacterial Strains, Plasmids, and Growth Conditions

Bacterial strains and plasmids used in this study are listed in Supplementary Table S1. *E. coli* LF82 is an AIEC strain isolated from a chronic ileal lesion of a CD patient (Boudeau et al., 1999). The LF82 *ΔmdtEF* strain, containing a deletion of the *mdtEF* genes, and MG1655 Δ*acr*AB, deleted of entire *acrAB* operon, have been constructed using the one-step method of gene inactivation (Datsenko and Wanner, 2000) by transforming LF82 pKD46 or MG1655 pKD46 with amplicon obtained using pKD13 as template and the oligo pairs EFF/EFR for *mdtEF* deletion and ABF-ABR for *acrAB* deletion (Supplementary Tables S1, S2).

Plasmid pGEF3 was obtained by cloning the *mdtEF* genes into pGIP7, a pACYC184 espression vector carrying the lacI^q^ gene and ptac promoter (Falconi et al., 2001; Supplementary Table S1). The *mdtEF* amplicon obtained using MG1655 as template and oligo pairs pACmdtEFF/pACmdtEFR (Supplementary Table S2) was digested with *Bam*HI and cloned into pGIP7 downstream the ptac promoter. Plasmid pGEF3 and deletion of *mtdEF* and *acrAB* genes have been verified by DNA sequencing (Biofab, Rome). Bacterial cells were grown aerobically in Luria-Bertani (LB) medium at 37°C. Congo Red at 0.01% was added to Trypticase soy agar to monitor the LF82 Congo Red phenotype. Antibiotics, and chemicals were used at the following concentrations: ampicillin 30 μg/ml, chloramphenicol 25 μg/ml, erythromycin 12.5 μg/ml or 25 μg/ml, kanamycin 30 μg/ml, and gentamicin 10 μg/ml or 100 μg/ml for infection procedures.

### General Procedures

DNA purification, restriction, cloning, plasmid transformation, and gel electrophoresis were carried out as previously described (De Carolis et al., 2011; Leuzzi et al., 2017). The oligonucleotide sequences, designed on the basis of the LF82 genome (Miquel et al., 2010) or MG1655 genome, are reported in Supplementary Table S2. PCR reactions were routinely performed using the DreamTaq DNA polymerase (Thermo Fisher Scientific) or, when required a higher fidelity of PCR product, the Ex taq DNA polymerase (Takara). DNA sequence data were compared to known nucleotide and protein sequences using the BLAST server (National Center for Biotechnology Information). Analysis of distribution of MDR EPs in the genome of other AIEC strains was performed using the EcoCyc database (Keseler et al., 2017).

### Cell Cultures and Infections

Both the human promonocytic U937 and the human monocytic THP-1 cell lines were grown in Roswell Park Memorial Institute (RPMI) 1640 (Gibco) medium containing 10% heat-inactivated fetal bovine serum (FBS; Euroclone), 0.05 IU/ml penicillin, and 0.05 IU/ml streptomycin (PS), referred to as RF10, at 37°C in a humidified 5% CO_2_ atmosphere. Before bacterial infection, both U937 and THP-1 cells were differentiated into macrophages. U937 cells were seeded in 6-well tissue culture plates (Falcon), at a density of 1.5 × 10^6^ cells/well, in RF10 supplemented with 80 nM phorbol myristate acetate (PMA; Sigma). After 2 days, PMA containing medium was removed and cells were left for further 4 days in RF10. Two hours before bacterial infection, RF10 was replaced with fresh RPMI. THP-1 monocytes were seeded in 6-well tissue culture plates (Falcon) at a density of 1.0 × 10^6^ cells/well in growth medium supplemented with 50 nM PMA. After 48 h, PMA containing medium was removed and cells left for further 24 h in RF10. Two hours before bacterial addition, RF10 was replaced with fresh RPMI. The human epithelial colorectal adenocarcinoma Caco-2 cell line was grown in Dulbecco minimal essential medium (DMEM; Gibco) containing 10% FBS and PS, referred to as DF10, at 37°C in a humidified 5% CO_2_ atmosphere. For bacterial infection, cells were seeded in 6-well tissue culture plates (Falcon) at a density of 4.0 × 10^5^ cells/well in growth medium. After 48 h, cells were serum-starved over-night in DMEM supplemented with 0.5% FBS and PS (DF0.5). Two hours before bacterial infection, DF0.5 was replaced with fresh DMEM without serum and antibiotics. Bacteria were added to the cell cultures at a multiplicity of infection of 100. After addition of bacteria, plates were centrifuged for 15 min at 750 × *g* and incubated 30 (U937 and THP1) or 45 min (Caco-2) at 37°C under 5% CO_2_ atmosphere to allow bacterial entry. Then extracellular bacteria were removed by three extensive washing with phosphate-buffered saline (PBS). This point was taken as time zero (T0). Fresh medium (RPMI or DMEM) containing gentamicin (100 μg/ml) was added to the other plates to kill extracellular bacteria, and infected cells were incubated at 37°C up to 4 or 5 h.

### RNA Isolation and Quantitative Real Time PCR

To monitor gene expression during host cell infection, one 6-well tissue culture plate was considered for each time point to maximize the yield of intracellular bacteria. Intracellular LF82 bacteria were recovered by lysing infected cells with 1% Triton X-100 (Sigma) for 5 min. Bacteria were diluted 1:2 with PBS to decrease Triton concentration before proceeding with RNA extraction (Di Martino et al., 2016). Two micrograms of total RNA were treated with DNAse I and retro-transcribed using the High Capacity cDNA Reverse Transcription Kit (Thermo Fischer Scientific). qRT-PCR was performed in a 30 μl reaction mix containing 3 μl cDNA using Power SYBR Green PCR Master Mix (Thermo Fischer Scientific) on a 7300 Real-Time PCR System (Thermo Fischer Scientific). At least three wells were run for each sample. Relative quantification was performed using the comparative cycle threshold (2^−ΔΔCt^) method (Livak and Schmittgen, 2001). Primers for the *nusA* transcript (endogenous control) and target transcripts were designed with the aid of the Primer Express software v2.0 (Thermo Fischer Scientific) and experimentally validated for suitability for the 2^−ΔΔCt^ method. All primers used are listed in Supplementary Table S2. In the case of EP systems consisting of more than one protein (AcrAB, EmrAB, EmrKY, MacAB, MdtJI, AcrEF, and MdtABC) encoded by genes clustered in a single operon, we monitored the transcription of the promoter-proximal gene.

### Live and Dead Assay

Intracellular dead bacteria were evaluated by staining the entire population with 4′,6-diamidino-2-phenylindole (DAPI; Sigma) and labeling dead cells with Propidium iodide (PI; Sigma). At the indicated time points, infected macrophages were lysed by adding 1% Triton X-100 in 1× PBS. The cell lysate was pelleted at 13,000 rpm for 5 min. The pellet containing intracellular bacteria was washed once in 1× PBS and suspended in 1x PBS containing 10 μg/ml DAPI and 15 μM PI. Samples were incubated 20 min at room temperature in the dark. Bacteria were centrifuged at 13,000 rpm for 5 min and washed once with 1× PBS. Pellet was resuspended in 20 μl 1X PBS containing 50% glycerol; 5 μl of the stained bacteria were added to the glass slide and overlaid with the coverslip for immediate observation and counting.

### Statistical Analyses

The statistical differences of the EP gene expression level between intracellular bacteria and bacteria grown in RPMI or DMEM were determined using Microsoft Excel by calculating the values of *p* derived from a one-way ANOVA. The statistical difference between the percentage of dead intracellular LF82 wt strain and the percentage of dead intracellular LF82 derivatives at each time point was determined by a two-tailed *t*-test.

## Results

### *In silico* Identification of the MDR EP Encoding Genes in AIEC LF82 Strain and Their Expression Profile During Infection of Host Cells

LF82 is a prototype strain of AIEC, isolated from CD patients (Boudeau et al., 1999). As previously reported (Miquel et al., 2010), the genome of LF82 contains 130 LF82 specific CDSs. Most of them (88.5%) are not found in any *E. coli* genome while the remaining percentage has no homology with genes identified in any pathogenic bacteria. Since among these LF82 specific CDSs there were no genes coding for MDR EPs, we searched in the genome of LF82 homologs for each of the 20 functional MDR EPs encoding operons described in *E. coli* K12 (Nishino and Yamaguchi, 2001; Kobayashi et al., 2006).

As shown in Table 1, 19 out of the 20 genetic systems encoding MDR EPs described in *E. coli* K12 MG1655 are present in LF82 genome. The *mdtM* gene is completely absent due to a severe rearrangement in the *mdtM-rpnD* locus as compared to MG1655 with the insertion of LF82 specific genes (Miquel et al., 2010). The remaining MDR EPs and their encoding genes show high homology with those of commensal *E. coli* K12 strain (Table 1). The pattern of functional MDR EPs of LF82 is the most common among those observed in highly invasive AIEC strains, isolated from CD patients, belonging to the B2 phylogroup (data not shown).

**Table 1:** Analysis of the multidrug efflux pump encoding genes present in the adherent-invasive *Escherichia coli* *(AIEC) LF82* genome.

Family	MG1655	LF82	DNA Sequence homology (%)^*^	Protein homology (%)^*^
ABC	*macAB*	+	98.17	98.65; 99.38
MFS	*bcr*	+	96.81	99.49
	*emrAB*	+	98.06	99.74; 99.80
	*emrD*	+	97.81	99.49
	*emrKY*	+	97.41	98.19; 100
	*fsr*	+	97.87	99.75
	*mdfA*	+	98.05	100
	*mdtG*	+	98.45	99.51
	*mdtH*	+	98.35	100
	*mdtL*	+	96.51	98.47
	*mdtM*	−	−	−
RND	*acrAB*	+	98.81	99.75; 100
	*acrD*	+	98.5	99.81
	*acrEF*	+	97.26	98.96; 99.52
	*cusB*	+	97.3	98.77
	*mdtABC*	+	95.35	98.80; 99.71; 98.83
	*mdtEF*	+	97.72	99.74; 99.42
SMR	*emrE*	+	98.8	99.09
	*mdtJI*	+	98.53	100; 100
MATE	*mdtK*	+	97.82	99.78

The 20 genetic systems encoding functional efflux pumps in *Escherichia coli* have been selected according to Nishino and Yamaguchi (2001). The comparative analysis between the genome of *E. coli* K-12 and AIEC LF82 strain was carried out using NCBI BLAST (http://blast.ncbi.nlm.nih.gov/Blast.cgi). The nucleotide sequences of each efflux pump encoding gene were obtained from EcoCyc (https://ecocyc.org).

+ Efflux pump encoding genes present in the LF82 genome. − Not present in the LF82 genome.

* Sequence and amino acid homology expressed as a percentage.

**Figure 1:**
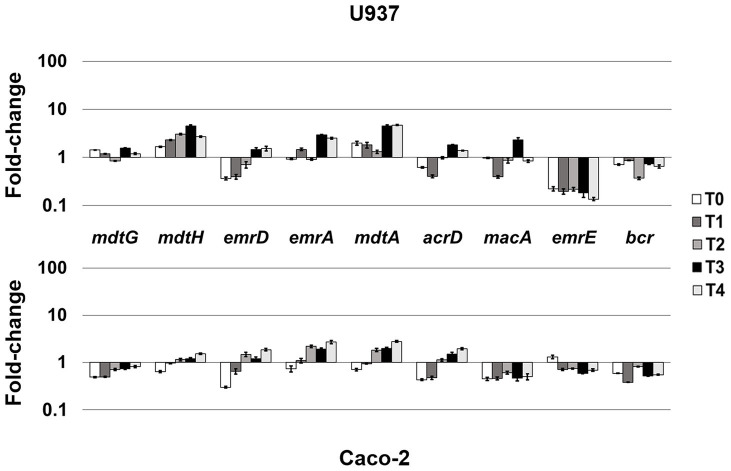
Mild-responsive MDR EPs to different cellular environments. Relative transcription of the EP encoding genes *mdtG*, *mdtH*, *emrD*, *emrA*, *mdtA*, *acrD*, *macA*, *emrE*, and *bcr* during LF82 infection of U937 and Caco-2 cells. Quantitative analysis of the transcripts was performed by qRT-PCR. Total RNA was extracted from LF82 bacteria at various time points p.i., from 0 h (corresponding to bacterial adhesion to and entry into target cells, T0) up to 4 h (T4) p.i. and from control bacteria grown in RPMI (U937) or DMEM (Caco-2). Each experiment was repeated three times and at least three wells were run for each sample. The results are shown as fold-change relative to the expression of each gene in control bacteria set to 1.0. A one-way ANOVA performed between intracellular bacteria and control bacteria yielded *p* < 0.01 for all the EP genes shown. Error bars represent SD.

**Figure 2:**
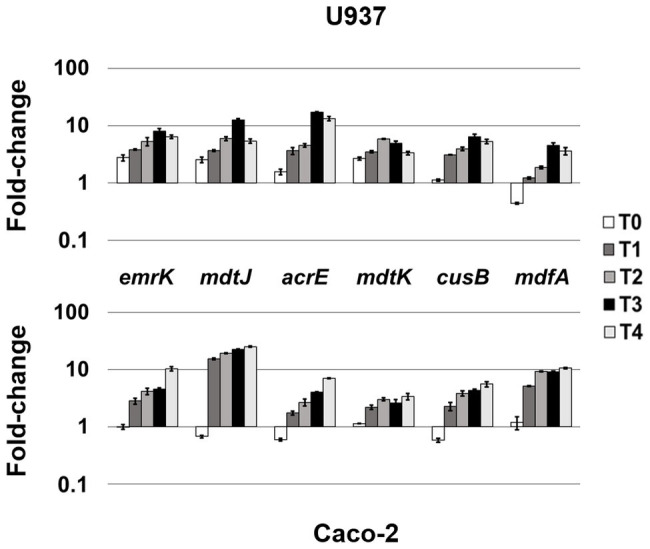
Highly-induced MDR EPs in both cellular environments. Relative transcription of the EP encoding genes *emrK*, *mdtJ*, *acrE*, *mdtK*, *cusB*, and *mdfA*, during LF82 infection of U937 and Caco-2 cells. Quantitative analysis of the transcripts was performed by qRT-PCR. Total RNA was extracted from LF82 bacteria at various time points p.i., from 0 h (corresponding to bacterial adhesion to and entry into target cells, T0) up to 4 h (T4) p.i. and from control bacteria grown in RPMI (U937) or DMEM (Caco-2). Each experiment was repeated three times and at least three wells were run for each sample. The results are shown as fold-change relative to the expression of each gene in control bacteria set to 1.00. A one-way ANOVA performed between intracellular bacteria and control bacteria yielded *p* < 0.01 for all the EP genes shown. Error bars represent SD.

**Figure 3:**
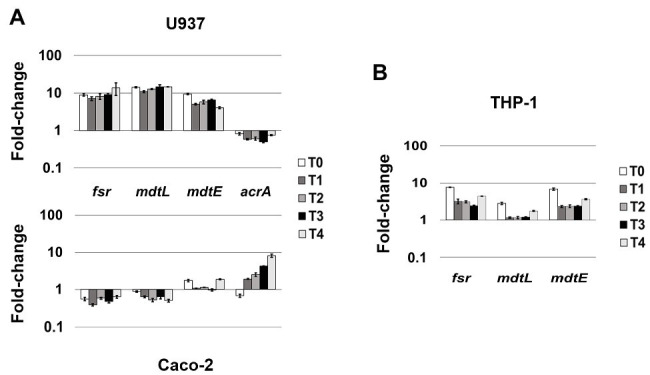
MDR EPs specifically responding to different cellular environment. **(A)** Relative transcription of the EP encoding genes *fsr*, *mdtL*, *mdtE*, and *acrA* during LF82 infection in U937 and Caco-2 cells. **(B)** Relative transcription of the EP encoding genes *fsr*, *mdtL*, and *mdtE* during the LF82 infection of THP-1 cells. Quantitative analysis of the transcripts was performed by qRT-PCR. Total RNA was extracted from LF82 bacteria at various time points p.i., from 0 h (corresponding to bacterial adhesion to and entry into target cells, T0) up to 4 h (T4) p.i. and from control bacteria grown in RPMI (U937 and THP-1) or DMEM (Caco-2). Each experiment was repeated three times and at least three wells were run for each sample. The results are shown as fold-change relative to the expression of each gene in control bacteria set to 1.00. A one-way ANOVA performed between intracellular bacteria and control bacteria yielded *p* < 0.01 for all the EP genes shown. Error bars represent SD.

LF82 is a pathogenic AIEC strain that is able to invade epithelial cells and survive and replicate widely in macrophages without inducing cell death (Boudeau et al., 1999; Glasser et al., 2001), suggesting that the bacterium is able to adapt and establish an equilibrium with the host cell. The expression of LF82 MDR EPs was therefore analyzed at early stage of the invasive process during the first 4 h of infection of human U937 monoblasts differentiated into macrophage-like cells and of Caco-2 epithelial cells. The global analysis by qRT-PCR shows that the expression of some of the conserved MDR EP encoding genes, such as *mdtG*, *mdtH*, *emrD*, *emrA* (MSF family), *mdtA*, *acrD* (RND family), and *macA* (ABC family), is weakly modified by host cell environment with few differences between the two cell types, or downregulated in both cell types, as for *emrE* (SMR family), which is heavily repressed particularly in macrophages, and *bcr* (MFS family; Figure 1). Concerning the other MDR EP genes conserved in LF82, results obtained by qRT-PCR analysis demonstrated that some of them are constantly upregulated during the infection of both cell types (Figure 2), while the expression of four genes, namely *fsr*, *mdtL*, *mdtEF*, and *acrA*, is regulated in a cell specific manner (Figure 3A).

### Some MDR EP Genes Are Induced Regardless of the Cell Type LF82 Is Infecting

The analysis of the transcriptional profile of all the genes encoding MDR EPs revealed that the genes *emrK* and *mdfA* (MFS family), *mdtJ* (SMR family), *acrE* and *cusB* (RND family), and *mdtK* (MATE family) are invariably induced during the infection of both U937 and Caco-2 cells, although at different extent (Figure 2). In particular, when LF82 strain infects macrophages, the *emrK*, *mdtJ*, *acrE*, and *mdtK* transcripts accumulate promptly after bacterium entry into the cell (T0), increasing throughout the infection period analyzed (Figure 2, upper panel). During the infection of epithelial cells, induction of the same genes is observed later, at 1 h post infection (p.i.), afterwards their expression follows a behavior similar to that observed in U937-derived macrophages, since the genes are strongly upregulated (Figure 2, lower panel). Particularly, the *mdtJ* transcript accumulates up to 25-fold at 4 h p.i., as compared to LF82 grown in DMEM medium (Figure 2, lower panel). The expression profile of the *cusB* gene is also quite comparable in the two cell types, with some differences at the very early stage of infection (T0) where the transcript levels keep unaltered in U937 cells, compared to control bacteria grown in RPMI medium, while decrease in LF82 infecting Caco-2 cells. Finally, among these group of EP genes, *mdfA* appears to be an exception, as its behavior at T0 is specular to that observed for the other EPs in the two cells types, being unchanged in epithelial cells, as compared to the control LF82, while downregulated in macrophages. The delay of induction observed in epithelial cells for virtually all these EP genes may be explained by the different mode of bacterium entry into the two cell types, direct phagocytosis by macrophages (Glasser et al., 2001) versus pilum-CEACAM6 interaction-dependent macropinocytosis (Glasser et al., 2001; Barnich et al., 2007), which might imply that bacteria face cellular response much earlier in macrophages than in epithelial cells.

Collectively, these data suggest that these MDR EP genes respond to stimuli common to both infected macrophages and epithelial cells and that their enhancement might be important for the overall invasive process of the bacterium.

### *fsr*, *mdtL*, *mdtEF*, and *acrA* MDR EP Genes Are Differentially Expressed During the Infection of Macrophages and Epithelial Cells

Unlike the genes described above, whose induction is independent from the cell type LF82 is infecting either macrophages or epithelial cells, the modulation of *fsr*, *mdtL*, *mdtEF*, and *acrA* EP genes appears to be driven by specific cell environment. Indeed, the transcript levels of the *fsr* and *mdtL*, encoding MDR EPs of the MFS family and *mdtE* encoding an MDR EP of the RND family, are strongly increased when LF82 invades U937-derived macrophages, as compared to LF82 grown in RPMI (Figure 3A). In particular, induction of the *frs*, *mdtL*, and *mdtE* genes occurs immediately as bacteria adhere and enter the host cell (T0; Figure 3A, upper panel). Conversely, the expression of these same genes is either downregulated, as in the case of *fsr* and *mdtL*, or virtually unchanged, as for *mdtE*, throughout the infection of epithelial cells (Figure 3A, lower panel). On the other way around, the *acrA* gene encoding the AcrAB EP (RND family) is specifically expressed when the LF82 invades epithelial cells. The transcription level of *acrA* gene increases over the time during Caco-2 cell infection (up to 8-fold), as compared to LF82 grown in DMEM (Figure 3A, lower panel). In contrast, the *acrA* gene is constantly downregulated during the infection of macrophages (Figure 3A, upper panel).

Overall, these findings suggest that the expression of some MDR EP genes in the LF82 AIEC strain represents a kind of signature mirroring the bacterial response to specific host-cell environment, either macrophagic or epithelial.

Resident macrophages in the intestinal tract are among the sentinels devoted at limiting systemic microbial dissemination and defending the organism from pathogen attacks. On the other hand, it is largely acknowledged that macrophages fail to restrict intracellular AIECs, which, in turn, survive and replicate inside them (Glasser et al., 2001; Vazeille et al., 2015; Palmela et al., 2018; Demarre et al., 2019). Hence, the specific induction of *frs*, *mdtL*, and *mdtE* genes observed in LF82 infecting U937 cells might be part of the bacterial strategies to counter the host-cell defenses. To strengthen this observation, the expression profile of these EP genes was also verified in THP-1 monocyte-derived into macrophages, widely used as model system to study the infection process of LF82 and other AIEC clinical strains (O’Brien et al., 2017; Demarre et al., 2019). Figure 3B shows that, although induced at lesser extent, the overall behavior of the three genes is quite similar to that observed in U937 cells. Noteworthy, the induction of these EP genes in LF82 is particularly significant as soon after the addition of bacteria to THP-1 cells, with *mdtL* being the least upregulated at T0 and almost unmodified at the other time points analyzed.

### MdtEF Contributes to the Survival of LF82 Inside Macrophages

**Figure 4:**
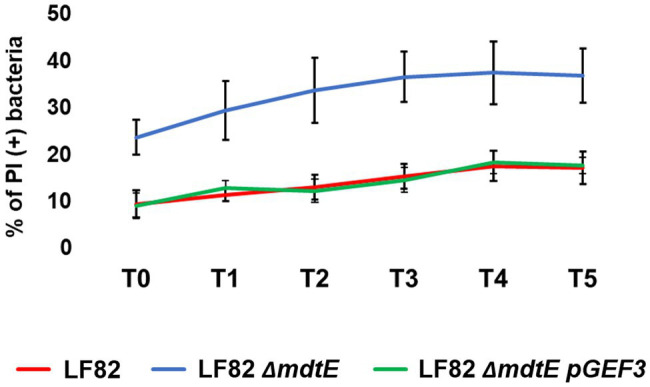
MdtEF pump favors survival of LF82 within macrophages. Intracellular bacteria were recovered from THP-1 differentiated macrophages at 0, 1, 2, 3, 4, and 5 h p.i. (referred to as T0, T1, T2, T3, T4, and T5, respectively) and soon after stained with DAPI/PI. The values are expressed as percentage of PI (+) dead bacteria relative to DAPI (+) bacteria. The results shown are the average of at least three independent experiments. A two-tailed *t* test performed between LF82 and LF82 Δ*mdtEF* or between LF82 Δ*mdtEF* pGEF3 and LF82 Δ*mdtEF* yielded a *p* < 0.01 at each time point while comparison between LF82 and LF82 Δ*mdtEF* pGEF3 yielded *p* > 0.1. Error bars represent SD.

More and more studies are addressing the emergent role of EPs in bacterial pathogenesis and virulence beyond that of conferring MDR (Piddock, 2006; Alcalde-Rico et al., 2016; Pasqua et al., 2019b). Recently, we have described how several EP genes are modulated in *S. flexneri* upon infection of macrophages and epithelial cells and how they can be functional for the macrophage invasive process (Pasqua et al., 2019a). *Shigella* and AIECs are two invasive *E. coli* pathotypes using very different virulence tools and strategies (Croxen et al., 2013). One of all is the outcome of macrophage infection, where *Shigella* rapidly exits the entry vacuole and eventually kills the cell (Ogawa and Sasakawa, 2006) while AIECs replicate inside the phagosome without inducing cell death (Glasser et al., 2001). Interestingly, during the evolution process toward intracellular lifestyle AIECs have conserved some genetic systems encoding MDR EPs, which are, instead, lost by *Shigella* (Pasqua et al., 2019a). Of note, *mdtEF* is among the MDR EP genes disrupted in *Shigella*, thus the specific induction seen in LF82 infecting macrophages may reflect, at least in part, the profound differences of the pathogenic mechanisms between the two pathogens. To get more insights on the value of the very early response of the LF82 inside macrophages, a LF82 *mdtEF* deletion mutant was generated by site specific mutagenesis (LF82 Δ*mdtEF*) and used to infect THP-1-derived macrophages. Parallel infections were performed with the wild type LF82 strain. Although AIECs persist and replicate inside macrophages, they are continuously under macrophage attack and a fraction of intracellular death bacteria is always present (Demarre et al., 2019). Hence, we asked whether lack of functional MdtEF could affect intramacrophagic LF82 survival. At the indicated time points, intracellular bacteria were recovered from infected macrophages and stained with PI and DAPI; the amount of PI positive dead LF82 and LF82 Δ*mdtEF* cells were evaluated under fluorescence microscopy. Data shown in Figure 4 confirm that a constant proportion, ranging from 10 to 17% of dead LF82 bacteria is detectable throughout the infection period analyzed. Interestingly, lack of MdtEF leads to a significant increase of bacteria that succumb to macrophage attack. Indeed, the percentage of PI positive LF82 Δ*mdtEF* shifts up 2–2.5-fold, depending on the time point considered, ranging from 24 to 38%. In order to assess the direct contribution of MdtEF in conferring intramacrophagic LF82 viability, we made attempts to restore the wild type phenotype in a LF82 Δ*mdtEF* by expressing a functional MdtEF protein complex. To this end, we constructed pGEF3 by cloning the *mdtEF* genes into the pGIP7 vector (Supplementary Table S1). Functionality of MdtEF pump encoded by pGEF3 was verified by testing the capability of pGEF3 to confer resistance to erythromycin in a *E. coli* K12 strain depleted of AcrAB, the major MDR EP (MG1655 Δ*acrAB*; Supplementary Table S3). Parallel infections of THP-1 macrophages were carried out with LF82, LF82 Δ*mdtEF*, LF82 Δ*mdtEF* pGIP7, or LF82 Δ*mdtEF* pGEF3. As exptected, LF82 Δ*mdtEF* pGIP7 strain behaves as the deleted mutant (data not shown). On the other way around, trans-complementation with pGEF3, encoding a functional MdtEF pump, completely restores the phenotype of LF82 Δ*mdtEF* strain. Indeed, Figure 4 shows that the proportion of dead LF82 Δ*mdtEF* pGEF3 bacteria is fully superimposable to that of dead wild type LF82 bacteria. The reduced viability of the LF82 Δ*mdtEF* indicates that the MdtEF EP participates in the bacterial response to the macrophage attack promoting LF82 survival.

Attempts to translate the viability phenotypes into a different number of colony forming bacteria failed due to the extremely high variability of growth curves obtained from different infection experiments both with the wild type and the mutant strains. This is in agreement with a recent study that well documents how intramacrophagic challenges induces a high LF82 phenotypic heterogeneity (Demarre et al., 2019), influencing the growth properties of intracellular bacteria. Altogether, these data indicate that increasing expression of the MdtEF encoding genes might be functional to activate a strategy for persisting in the harsh environment of the macrophage phagolysosome.

## Discussion

In the present study, we analyzed the differential expression of genes encoding the MDR EPs of the AIEC LF82 strain during the first steps of its intracellular life. We found that some of these genes are highly and specifically activated during infection of macrophages while others are specifically induced when bacteria infect epithelial cells. Among MDR EPs significantly activated in the macrophage environment, we have focused on MdtEF, an EP belonging to the RND family, and we were able to show that its expression is linked to reduced mortality of LF82 within macrophages.

AIEC are a peculiar group of enteropathogenic *E. coli*, very frequently found in CD patients, able to colonize the intestinal epithelial cells and to actively replicate in macrophages (Darfeuille-Michaud, 2002; Palmela et al., 2018; Shaler et al., 2019). Adhesion of AIEC on ileal enterocytes is mediated by the interaction of bacterial type1 pili with the host cell receptor CEACAM6. The high expression of this receptor at the apical surface of enterocytes in CD patients accounts for the massive presence of AIEC in their intestinal epithelia (Barnich et al., 2007). In addition to invasion of intestinal epithelial cells, AIEC are able to translocate through the epithelium and gain access to the macrophages. AIEC replicate extensively within macrophage phagolysosomes without triggering cell death. Continuous replication of AIEC in infected macrophages results in secretion of high levels of TNF-α, causing strong intestinal inflammation in CD patients (Glasser et al., 2001). The intravacuolar microenvironment of macrophage phagolysosomes not only protects bacteria from autophagy (Lapaquette et al., 2010) but also favors full expression of the virulence phenotype and active replication (Bringer et al., 2006). Recently, it has been shown that persistence within macrophages exposes AIEC LF82 to several stresses, resulting in a complex bacterial response that promotes the formation of non-growing and antibiotic tolerant variants (Demarre et al., 2019).

A growing body of studies suggests that the functional significance of MDR EPs goes beyond their capability to extrude a wide range of antibiotics as these pumps are also involved in relevant aspects of bacterial cell physiology, including interactions with host cells (Alcalde-Rico et al., 2016; Du et al., 2018; Pasqua et al., 2019b). While in other enteroinvasive bacteria, such as *Salmonella* and *Shigella*, it was shown that MDR EPs contribute to the bacterial survival within the host cells (Buckley et al., 2006; Pasqua et al., 2019a), no data are available for AIEC strains. LF82 is a reference AIEC strain isolated from CD patients (Boudeau et al., 1999) that has been widely used to investigate bacteria-host interactions. As compared to the commensal *E. coli* K12, we observed that the LF82 genome contains all functional MDR EPs previously described with the exception of MtdM, whose operon has been disrupted by the insertion of AIEC specific genes. We found that the expression of many of the MDR EP genes conserved in LF82 is strongly modified by intracellular environment, suggesting a potential involvement of these systems in the infection process also in AIEC. Notably, some MDR EPs are invariably induced regardless of the cell type LF82 is infecting, indicating that the bacterium is subjected to common stimuli in both U937-derived macrophages and Caco-2 epithelial cells (Figure 2). Among these MDR EPs, EmrKY is of particular interest. Indeed, as we have previously reported, in *Shigella*, EmrKY is selectively expressed in macrophages and inhibited in epithelial cells (Pasqua et al., 2019a), while here we found that in AIEC this EP is actively transcribed in both cell types throughout the analyzed infection period. As demonstrated before the acidic cytoplasmic pH determined by *Shigella* infection of macrophages is responsible for EmrKY induction (Pasqua et al., 2019a). AIEC and *Shigella* both belong to the *E. coli* species and share the capacity to invade macrophages and epithelial cells (Croxen et al., 2013). However, their infection strategy greatly differs, as *Shigella* rapidly escapes from phagolysosome upon infection of both macrophagic and epithelial cells and multiplies in the cytoplasm (Schroeder and Hilbi, 2008), while AIEC remain and multiply inside the phagolysosome or the late endosome in macrophages and epithelial cells, respectively (Glasser et al., 2001; Lapaquette et al., 2010). Hence, the induction of *emrK* gene in LF82 during the infection of both macrophages and epithelial cells is likely because AIEC are continuously exposed to an acidic environment, such as that of phagolysosome or late endosome, in both cell types.

In addition to the LF82 EPs being highly induced by both cell environments, we observed that the expression of few EP genes is specifically induced when the bacterium infects U937-derived macrophages or Caco-2 epithelial cells. Particularly, the gene *acrA*, encoding the periplasmic component of the AcrAB efflux pump, is upregulated when LF82 strain invades epithelial cells, while is downregulated in macrophages. The AcrAB efflux pump belongs to the RND family and it is known as the major *E. coli* MDR efflux system (Nishino and Yamaguchi, 2001); therefore, its downregulation in the macrophage environment might be complemented by overexpression of other pumps that could fulfill, at least in part, the same function. Accordingly, the expression levels of the *fsr* and *mdtL*, encoding MDR EPs of the MFS family and *mdtE* encoding an EP of the RND family, are strongly increased when LF82 invades U937-derived macrophages, while lowered when Caco-2 cells are infected. Whether the activity associated to these MDR EPs can complement the downregulation of AcrAB has still to be determined. However, the specular expression profile of this group of EPs in LF82 infecting either macrophages or epithelial cells appears intriguing. In this context, it is worth mentioning that in *Salmonella* Typhimurium the silencing of *acrA* or *acrF*, which encode the transporter component of two RND EPs, increases the expression of *acrD*, a gene encoding another RND EP (Eaves et al., 2004).

The induction of Fsr and MdtE appears to be a common response of LF82 to macrophagic environment as we find them upregulated during the infection of a different monocytic cell line, the widely used THP-1-derived macrophages (Figure 3B). This observation strongly supports the hypothesis that these EPs might be part of the pathogenic strategy of LF82, favoring survival and multiplication inside macrophages.

From an evolutionary point of view, MdtEF is of particular interest, as it is among the MDR EPs lost by *Shigella* (Pasqua et al., 2019a), likely because unnecessary during the intracellular life.

MdtEF forms a tripartite EP with the common outer membrane channel TolC. In *E. coli* K12, the overexpression of MdtEF confers resistance to a large panel of antibiotics and toxic compounds, including fatty acids and sodium deoxycholate (Nishino and Yamaguchi, 2001). The *mtdEF* genes are located downstream the *gadE* gene, which encodes the key regulator of a major acid resistance system. Transcriptional regulation from the *gadE* promoter has been extensively studied and involves, among others, the response regulators ArcA, EvgA, and PhoP, the nucleoid associated protein H-NS, the sigma factor RpoS and the regulatory sRNA DsrA (Nishino et al., 2011; Zhang et al., 2011). The expression of *mdtEF* genes is induced by various physiological and environmental stimuli, e.g., stationary growth phase or presence of indole and acetylglucosamine, as well as by oxygen and acid stress (Deng et al., 2013). While it has been shown that MdtEF protects the *E. coli* cells from nitrosative damage during anaerobic conditions (Zhang et al., 2011), its potential role in pathogenicity has not yet been fully explored. The data we have obtained on the viability phenotype of intracellular LF82 bacteria expressing or not the MdtEF pump indicate that this EP, probably because of its capacity to extrude a large panel of compounds, might favor the intravacuolar survival of LF82, likely pumping out toxic metabolites. The capability of the *mdtEF* genes expressed by the pGEF3 plasmid to fully restore the intracellular viability in LF82 Δ*mdtEF* strain further confirms the contribution of this EP to the LF82 fitness in the harsh macrophage niche. It has been recently reported (Demarre et al., 2019) that, inside the macrophages, LF82 strain rapidly activates the response to acid and oxidative stress, to envelope alterations and to the lack of important nutrients. The inclusion of *mdtEF* operon in a complex regulatory network able to perceive and respond to the severe stresses from macrophage phagolysosome allows the rapid induction of its transcription soon after the bacterial entry into the cell. The prompt activation of MdtEF encoding genes would provide a high abundance of this pump, promoting a quick defensive response to the hostile phagolysosome environment.

Further studies will be necessary to clearly define the contribution of other EPs in the interactions of LF82 with the host cells. Up to now, the role we disclosed for MdtEF in improving survival inside the stressing environment of the macrophagic phagosome represents the first evidence of the involvement of an MDR EP in the infection strategy of an AIEC invasive strain. This further highlights the critical role of the RND systems in both MDR and virulence.

## Data Availability Statement

The raw data supporting the conclusions of this article will be made available by the authors, without undue reservation.

## Author Contributions

GF, MG, MP, GP, and BC conceived and designed the experiments. GF, MP, and MG performed the experiments. GF, MP, GP, and MG analyzed the data. BC, MG, and GP contributed reagents, materials, and analysis tools. GF, BC, MG, and GP wrote the paper. All authors contributed to the article and approved the submitted version.

### Conflict of Interest

The authors declare that the research was conducted in the absence of any commercial or financial relationships that could be construed as a potential conflict of interest.

## Abbreviations

ABC: ATP binding cassette
AbgT: p-aminobenzoyl-glutamate transporter
AIEC: Adherent invasive *E. coli*
CD: Crohn disease
DAPI: 4',6-diamidino-2-phenylindole
EP: Efflux pump
MDR: Multidrug resistance
MFS: Major facilitator superfamily
MATE: Multidrug and toxic compound efflux
PACE: Proteobacterial antimicrobial compound efflux
p.i.: Post infection
PI: Propidium iodide
RND: Resistance nodulation division
TNF: Tumor necrosis factor.
