# Supplementary material for: Expression Profile of Multidrug Resistance Efflux Pumps During Intracellular Life of Adherent-Invasive Escherichia coli Strain LF82
Source: Front Microbiol. 2020 Aug 17;11:1935. doi: 10.3389/fmicb.2020.01935 (PMC7462009; doi:10.3389/fmicb.2020.01935)
Supplement: Supplementary file 1 [file Data_Sheet_1.DOCX]

**Table S1 Strains and plasmid used in this study**

| **Strains** | **Characteristic** | **Source/References** |
| --- | --- | --- |
| LF82 | AIEC strain isolated from a chronic ileal lesion of CD patient | Boudeau et al., 1999 |
| LF82 *∆mdtEF* | LF82 derivative strain defective in *mdtEF* genes, Km^R^ | This study |
| MG1655 *∆acrAB* | MG1655 derivate strain defective in *acrAB* operon | This study |
| **Plasmids** |  |  |
| pKD13 | Kan^R^-containing plasmid, template for PCR | Datsenko and Wanner, 2000 |
| pKD46 | Temperature sensitive replicon that carried bacteriophage λ red genes (γ, β and exo) under control of arabinose inducible P_araBAD_ promoter, Gm^R^ | Datsenko and Wanner, 2000 |
| pGIP7 | pACYC184-derived vector carrying lacI^q^-lac promoter region,Cm^R^ | Falconi et al., 2001 |
| pGEF3 | pGIP7 derivative plasmid carrying the *mdtEF* genes, Cm^R^ | This study |
