# Supplementary material for: Expression Profile of Multidrug Resistance Efflux Pumps During Intracellular Life of Adherent-Invasive Escherichia coli Strain LF82
Source: Front Microbiol. 2020 Aug 17;11:1935. doi: 10.3389/fmicb.2020.01935 (PMC7462009; doi:10.3389/fmicb.2020.01935)
Supplement: Supplementary file 2 [file Data_Sheet_2.DOCX]

**Table S2 Oligonucleotides used in this study**

| **Chromosomal mutations** | |
| --- | --- |
| **Name** | **5’-3’ sequences** |
| EFF | AATGTGTTGAGCGAATTGCCCGGTAGAACCGTTCCTGTGTAGGCTGGAGCTGCTTC |
| EFR | AACGAAAAAGACCGGAACGAAGTAAATTGCCAGCACATTCCGGGGATCCGTCGA |
| ABF | ACTTTTGACCATTGACCAATTTGAAATCGGACACTCGAGGTTTACATATGTGTAGGCTGGAGCTGCTTC |
| ABR | CATAAAAAAGGCCGCTTACGCGGCCTTAGTGATTACACGTTGTATCAATG ATTGGGGATCCGTCGACC |
| **Construction of plasmids** | |
| **Name** | **5’-3’ sequences** |
| pACmdtEFF | NNNGGATCCATGAACAGAAGAAGAAAGCTGTT |
| pACmdtEFR | NNNGGATCCCCAGGCGGTATCGGTATTTTC |
| **qRT-PCR** | |
| **Name** | **5’-3’ sequences** |
| acrAF | GTCTATCACCCTACGCGCTATCTT |
| acrAR | GCGCGCACGAACATACC |
| acrDF | AGCTATCGCCCTGGTTTTCCT |
| acrDR | AAGGTTCCCATCAACACCACC |
| acrEF | TATTGTGAAAACGGCCCCG |
| acrER | TCAGTACGATCCCGCTAACCTG |
| cusBF | CAAAGATAACGTGGTCGCGAA |
| cusBR | CGTAAACTGCGAGGCATCTTTC |
| emrAF | GCGAATATTGAGGTGCAGAAAA |
| emrAR | GGCACACGGCGGTTGTA |
| emrDF | GTGGATCCCCGACTGGTTT |
| emrDR | CCCGGCACCGAAAAAGA |
| emrEF | GGTTATTAGCTCAGACGCTGGC |
| emrER | TGGCCGAAAAATCCCCAT |
| emrKF | TGACCAATCCGTACATCCGTG |
| emrKR | ACAGTGAGTCCCGGACAATCAC |
| fsrF | TGGTGTTGGCGCAAATCA |
| fsrR | TCGTCGCTTTGGGTTTTCC |
| macAF | GACGCCGGAAAAGATTAACGA |
| macAR | TATGCACTTGCGCGGTCAT |
| mdfAF | ATTCACCCTGTTGCGCTTCTT |
| mdfAR | CTTGATACAAACGGCCTCTTCG |
| mdtAF | GGATGATGCGCTTTTCCCTAAT |
| mdtAR | TCGTTGC CCATTTGCAGTG |
| mdtEF | ACCGCCTGCGATGACAAAT |
| mdtER | GGCAATTCGCTCAACACATTG |
| mdtGF | CGGTATTGTCTTCAGCATTACATTTT |
| mdtGR | GGCGAGTCCACCCCAAA |
| mdtHF | AAAAGTTGTTGCAGGCCGC |
| mdtHR | GCTGGAGTGAAAAGCATTTTCG |
| mdtJF | CCCGGCAATTTTCATCAGC |
| mdtJR | CGCCGTTAAGAAAATCGCCT |
| mdtKF | CCGGTTATCGCGCAATTAAAT |
| mdtKR | GAAACCTTGTCGCACCTGATG |
| mdtLF | TTCTGGACGATGGCAACGAT |
| mdtLR | CGTGGTTTGTCCGAAGTTGTG |
| nusAF | TGAAGCCGCACGTTATGAAG |
| nusAR | TCAACGTAATCGCCCAGGTT |
