# Supplementary material for: Expression Profile of Multidrug Resistance Efflux Pumps During Intracellular Life of Adherent-Invasive Escherichia coli Strain LF82
Source: Front Microbiol. 2020 Aug 17;11:1935. doi: 10.3389/fmicb.2020.01935 (PMC7462009; doi:10.3389/fmicb.2020.01935)
Supplement: Supplementary file 3 [file Data_Sheet_3.DOCX]

**Table S3. Functionality of the MdtEF pump expressed by pGEF3**

| **Strain** | **MG1655** | **MG1655 Δ*acrAB*** | **MG1655 ΔacrAB pGIP7** | **MG1655 *ΔacrAB* pGEF3** |
| --- | --- | --- | --- | --- |
| Erytromycin 12.5 µg/ml | R | S | S | R |
| Erytromycin 25 µg/ml | R | S | S | R |

S: sensitive strain; R: resistant strain
